# Natural Fermentation of Cowpea *(Vigna unguiculata)* Flour Improves the Nutritive Utilization of Indispensable Amino Acids and Phosphorus by Growing Rats

**DOI:** 10.3390/nu12082186

**Published:** 2020-07-23

**Authors:** Garyfallia Kapravelou, Rosario Martínez, Jole Martino, Jesus M. Porres, Ignacio Fernández-Fígares

**Affiliations:** 1 Department of Physiology, Institute of Nutrition and Food Technology, Universidad de Granada, Campus Universitario Cartuja s/n, 18071 Granada, Spain; kapravelou@ugr.es (G.K.); rosariomz@ugr.es (R.M.); jolemartino78@gmail.com (J.M.); 2 Department of Physiology and Biochemistry of Animal Nutrition, Estación Experimental del Zaidín (EEZ), CSIC, Profesor Albareda 1, 18008 Granada, Spain; ifigares@eez.csic.es

**Keywords:** *Vigna unguiculata*, fermentation, digestibility, amino acids, mineral bioavailability, rat

## Abstract

Cowpea *(Vigna unguiculata)* is among the most cultivated legumes, with interesting agronomic and environmental properties, and great potential as a nutritious food. The nutritional value of cowpea can be improved by technological processing. In this study, we showed that natural fermentation improved bioavailability of protein, amino acids, and dietary essential minerals from cowpea in growing rats, thus strengthening its potential value as functional food or food supplement. Forty Wistar albino rats (48 ± 1.8 g), were fed one of four experimental diets (*n* = 10 rats per diet): casein, raw cowpea, fermented cowpea or fermented and autoclaved cowpea. Despite lower growth indices of raw and fermented cowpea protein (PER, FTI) than casein, fermentation enhanced apparent digestibility of arginine, leucine, lysine, methionine, phenylalanine, tyrosine, and valine, and true digestibility of essential amino acids, except for tyrosine and valine, compared to raw cowpea. On the other hand, autoclaving of fermented cowpea flour decreased apparent, as did true digestibility of sulfur amino acids. Regarding the nutritive utilization of dietary essential minerals, *Vigna unguiculata* was a good source of available P, Mg, and K, while fermentation significantly improved the availability of P. Overall, cowpea was a good source of digestible essential amino acids and minerals and fermentation significantly improved its nutritional value that was not further enhanced by autoclaving.

## 1. Introduction

Legumes are important dietary sources of essential nutrients for human and animal nutrition, such as protein, complex carbohydrates, vitamins, and minerals. However, they also contain non nutritional compounds (α-galactoside oligosaccharides, trypsin inhibitors, lectins, polyphenols, or phytic acid) that can interfere with the nutritional value of legumes but at the same time potentiate/increase their value as functional foods [1]. In addition to the presence of antinutritional components, legumes and other plant-based protein sources have a lower potential to boost protein metabolism and retention rate at the whole body or muscle levels in animals or humans when compared to animal-based proteins. They also exhibit lower digestibility and incomplete profile of essential and biologically-active amino acids, such as leucine, lysine, or sulfur-containing amino acids than animal proteins, which can limit in vivo protein synthesis.

When processed, legumes increase their palatability and nutritive utilization. Processing may also contribute to enhancing the health benefits attributed to legume consumption. Soaking, cooking, germination, and fermentation are amongst the most effective and widely used processing methods, improving the nutritional [2,3] and functional value of legumes [4,5]. The development of novel legume-derived foods that exhibit high nutritional value and efficient action at promoting health has gained increasing attention in recent years. In this regard, cowpea (*Vigna unguiculata*) is a widely cultivated legume in Asia, Central and South America, and Africa, where it is an important ingredient of several dishes that involve a different degree of culinary and technological processing [6,7]. Furthermore, Phillips et al. [8] and Ayogu et al. [9] discussed its potential to take part in the design and preparation of a variety of new food products, such as snacks, weaning foods, wheat-based cookies, or fortified traditional foods.

The cowpea has a substantial content of protein, complex carbohydrates, minerals, and vitamins, but also exhibits appreciable levels of phytic acid, tannins, α-galactoside oligosaccharides, and trypsin inhibitor activity that may interfere with its nutritive utilization. Fermentation of legumes contributes in developing flavor, aroma, and texture, and enhances the nutritive value by improving the density and availability of nutrients through the destruction of antinutritional factors, pre-digestion of certain food components, and synthesis of promoters for absorption [10,11,12,13,14]. Furthermore, this biotechnological process has the capacity to cause significant modifications in legume-derived bioactive components, therefore increasing the health benefits of cowpea consumption [4,15]. Heat treatments are also widely used to improve the nutritional value of legumes due to its action on heat-labile non-nutritional components including proteolytic inhibitors, thus improving protein digestibility [16,17]. Such treatments may be combined with other technological processes, such as germination or fermentation, to potentiate their beneficial actions on nutrient composition and bioavailability [12,18].

To our knowledge, little is known regarding the digestibility of amino acids in cowpea and how the availability of protein and essential minerals is affected by the biotechnological processes of fermentation and its combination with other technological processing. Therefore, the objective of this study was to assess whether natural fermentation or its combination with autoclaving was able to improve nutritive utilization of protein, amino acids, and essential minerals like P, Ca, Mg, or K from cowpea. To evaluate the nutritional potential of raw and fermented cowpea protein, we used a growing rat experimental model and a well-stablished casein control reference protein with high nutritional value consistently described in the literature [19,20].

## 2. Materials and Methods

### 2.1. Plant Material, Fermentation and Thermal Treatment of the Fermented Flours

Cowpea seeds (*Vigna unguiculata* L. var. carilla, La Pedriza, Cadiz, Spain) purchased at a local market were washed with distilled water, dried on a stove oven at 55 °C for 24 h, and ground to a fine powder (0.18 mm sieve) for chemical analysis and diet preparation without any other prior treatment. For fermentation, a suspension of raw cowpea flour in sterile distilled water (300 g·L^−1^) was prepared and allowed to ferment naturally (37 °C, 48 h) with the microorganisms present in the seed without aeration in a 5 L stirred fermenter (Infors ISF-100; Infors, Bottmingen, Switzerland) at 150 rpm. After fermentation, the samples were collected and freeze-dried. The thermal treatment of the fermented cowpea flour consisted of autoclaving for 15 min at 121 °C, 1 atm.

### 2.2. Animal Trial

**Figure 1:**
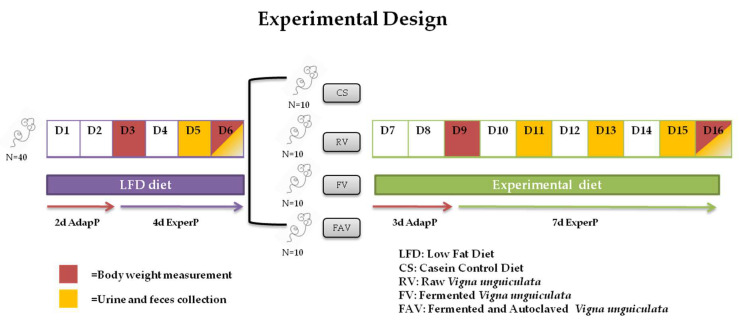
Experimental design of the study. D1–D16, correspond to day 1 to day 16 of the experiment; AdapP: Adaptation Period; ExperP: Experimental Period.

A total of 40 (3 weeks old) Wistar albino rats, were randomly distributed into four experimental groups (48 ± 1.8 g BW; five males and five females per group). Different diets were administered in each group: casein (control), raw *Vigna unguiculata* (RV), fermented *Vigna unguiculata* (FV), and fermented and autoclaved *Vigna unguiculata* (FAV). Diets were formulated following the recommendations of the American Institute of Nutrition [21] to meet the nutrient requirements of growing rats [22]; all diets were supplemented with 5 g·kg^−1^ methionine, first limiting amino acid in legumes, to avoid an amino acid imbalance. Ingredients and nutrient composition of the diets are shown in Table 1 and Table 2. Specifically, the target crude protein content of diets was 120 g·kg^−1^ (12%). To meet this percentage of protein, 500 g of cowpea flour was added to the experimental diets and 136 g of casein in the control diet. Moreover, since dietary fiber is higher in cowpea flour than in casein [23], a combination of insoluble (cellulose, agar, oat xylan, potato starch, and lignin) and soluble fiber (citrus pectin) was supplemented to the control casein diet. Moreover, the amounts of Ca, P, Mg, and K provided by the cowpea flours [2] were taken into consideration for their final concentration present in the diet; additional amounts of these minerals were supplied as Ca-citrate, CaHPO_4_ and K-citrate accordingly, whereas no Mg was added to the cowpea-containing diets. The rest of the minerals were included in the AIN-93G mineral premix. To estimate endogenous protein secretion by Wistar rats, an experimental bioassay using diets with different protein concentrations (4%, 8%, 12%, 16%, and 20%) was previously performed, showing that similar values of protein excretion were detected for both 0% and 4% protein level (Figure 1, Appendix A). Since a zero-protein level could be physiologically inadequate for the correct animal growth, we decided to include an isoenergetic low protein diet (LPD4%, 40 g·kg^−1^) to estimate the endogenous protein excretion of each animal that would serve as its own control, prior to the consumption of the experimental diets.

The animals were divided into four groups of 10 rats each (5 males and 5 females) per group and kept in metabolic cages designed for separate collection of feces and urine (Figure 1). The cages were placed in a well-ventilated, thermostatically controlled (21 ± 2 °C) room with 12 h light/dark periods (09:00/21:00). Prior to experimental diet consumption, all the animals consumed ad libitum LPD4% diet for 6 days (2-day adaptation and 4-day experimental) in order to calculate endogenous fecal losses of protein and amino acids. Subsequently, each group consumed ad libitum one of the experimental diets for 10 days (3-day adaptation and 7-day experimental). Separate collection of both feces and urine was carried out on alternate days throughout the experimental period. Fecal samples were freeze-dried and stored at −20 °C and urine samples were collected under acidic conditions and kept refrigerated until analysis. Daily food intake was determined by weighing the amounts of diet given, refused, and spilled. Body weight was recorded on days 3 and 6, as well as days 9 and 16, corresponding to the low-protein and experimental periods, respectively. Throughout the trial, all the rats had free access to double distilled water. At the end of the experiment, rats were deprived of food for 12 h, anaesthetized with pentobarbital, and sacrificed. All experiments were undertaken according to Directional Guides Related to Animal Housing and Care (Directive 2010/63/EU of the European Parliament and of the Council on the protection of animals used for scientific purposes) and all procedures were approved by the Animal Experimentation Ethics Committee of the University of Granada (project reference P09-AGR-4658).

### 2.3. Chemical Analysis

All analyses were performed in duplicate. Dry matter (ID 934.01) and nitrogen (ID 984.13) were determined according to the Association of Official Analytical Chemists (Association of Official Analytical Chemists 2000). Amino acids were determined in diets and feces by HPLC as previously described [24]. Ash content of diet, feces, and the different tissues assayed (blood, femur, and longissimus dorsi muscle) was measured by calcination at 500 °C to a constant weight. Samples of ashed material were dissolved in 6N HCl before analysis. Calcium and magnesium contents were determined by atomic absorption spectrophotometry using a Perkin-Elmer AAnalyst 300 spectrophotometer. Potassium content was determined by atomic emission spectrophotometry using a Perkin-Elmer AAnalyst 300 spectrophotometer. Total phosphorus was measured spectrophotometrically using the technique described by Chen et al. [25].

### 2.4. Biological Indices

The following indices and parameters for food intake and growth performance were used for each experimental group: weekly intake (expressed as dry matter), weight gain, protein efficiency ratio (PER; weight gain/protein intake), and food transformation index (FTI; total food intake/increase in body weight).

To determine digestive utilization of protein, amino acids and minerals, key parameters/indices were calculated as follows: apparent fecal digestibility (AFD) of protein, amino acids, and minerals was obtained using the formula AFD=[1−(Sf/Sd)], where *Sf* and *Sd* indicate amount of the nutrient in feces and diet, respectively. True fecal digestibility (TFD) of amino acids and protein was obtained from the formula TFD=[1−(Sf−Se/Sd)] where *Sf*, *Sd*, and *Se* indicate nutrient amount in feces, diet, and endogenous excretion, respectively.

Metabolic utilization of protein and minerals was assessed using the following parameters and indices:

(1) Retention (balance)=I−(F+U)

(2) Retention to absorption index (R/A)={I−(F+U)/(I−F)} 

where *I* = intake, *F* = fecal excretion, and *U* = urinary excretion.

True retained protein and R/A were calculated by subtracting the endogenous fecal and urinary losses from the total fecal and urinary values using the following formula:

(3) True Retained Protein = I−[(F−Fe)+(U−Ue)]

(4) True R/A ={[I−(F−Fe)−(U−Ue)]/[I−(F−Fe)]}

where *I* = intake, *F* = fecal excretion, *Fe* = endogenous fecal excretion, *U* = urinary excretion, and *Ue* = endogenous urinary excretion.

Amino acid ratios were calculated for essential amino acids:

(5) (Amino acid in test protein/Amino acid in reference protein)

The pattern of amino acid requirement for preschool children (2–5 years) was used as the reference protein (WHO/FAO/UNU, 2007). The lowest amino acid ratio, corresponding to the limiting amino acid, was reported as the chemical score (CS). The protein digestibility-corrected amino acid score (PDCAAS) was calculated from CS and TFD of protein:

(6) $$\frac{\mathrm{mg}\;\mathrm{amino}\;\mathrm{acid}\;\mathrm{in}\;1\;g\;\mathrm{test}\;\mathrm{protein}}{\mathrm{mg}\;\mathrm{of}\;\mathrm{amino}\;\mathrm{acid}\;\mathrm{in}\;\mathrm{requirement}\;\mathrm{pattern}}\times \mathrm{TFD}$$

### 2.5. Statistical Analysis

The results are expressed as means (*n* = 10 rats/treatment) and individual standard error. Statistical comparison of the experimental groups was done by one-way ANOVA test, applying a significance level of *p* < 0.05. Comparisons between each group were made using Tukey’s test when the ANOVA results were statistically significant. Assumptions of the test included a normal distribution of the data, equal variances, and randomization of the independent sample groups. Normality and homogeneity of variances were checked with the Shapiro–Wilk and Levene test, respectively. The statistical analysis was carried out using the computer software package Statgraphics Centurion XVI (Stat Point Technologies, Inc. Warrenton, VA, USA).

## 3. Results

### 3.1. Chemical Analysis

Chemical composition of diets is shown in Table 1. Crude protein (N × 6.25) content was 130 and 119 g·kg^−1^ for control and cowpea diet, respectively. All the experimental diets provided sufficient amounts of Ca, P, Mg, and K to meet the nutrient requirements of growing rats. The amino acid contents (g·kg^−1^ dry matter) in the foodstuffs together with the amino acid profiles of the protein (g/16 g N) are shown in Table 2, compared to egg protein as reference protein [26]. Proteins from cowpea had good levels of indispensable amino acids Phe + Tyr, Arg, Leu, and Lys, and of Glu + Gln among dispensable amino acids. Asp, Glu, Leu, Lys, and Arg were the amino acids with greater concentration in cowpea diets, accounting for 46% of the total amino acidic nitrogen while in the casein diet Glu, Lys, Leu, and Pro accounted for 47%. 

### 3.2. Biological Analysis

#### 3.2.1. General Growth Parameters and Nutritive Utilization of N

**Figure 2:**
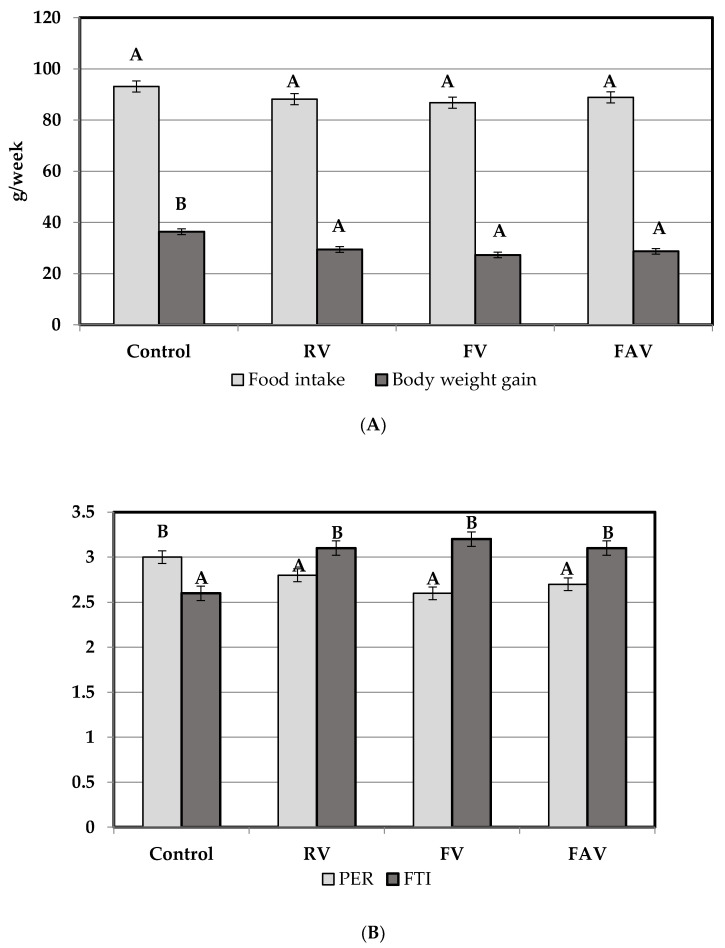
Effects of raw and fermented *Vigna unguiculata* on food intake and growth parameters of rats. (**A**) Food intake and body weight in the different experimental diets. Control: casein diet, RV: raw cowpea diet; FV: fermented cowpea diet; FAV: fermented and autoclaved cowpea diet. Different letters above columns indicate significant differences (*p* < 0.05). (**B**) Growth parameters in the different experimental diets. Control: casein diet; RV: raw cowpea diet; FV: fermented cowpea diet; FAV: fermented and autoclaved cowpea diet. PER: Protein Efficiency Ratio (weight gain, grams per day/protein intake, grams per day); FTI: Food Transformation Index (total intake, grams dry matter per day/increase in body weight). Different letters above columns indicate significant differences (*p* < 0.05).

There were no differences in food intake among dietary groups (*p* > 0.05; Figure 2A). Body weight gain and PER were higher in animals fed control diet (*p* < 0.05) compared to those fed the different cowpea diets among which no differences were found. In contrast, FTI was significantly higher in the cowpea-fed vs. control-fed animals (Figure 2B). On the other hand, rats fed control diet had greater protein intake, better growth parameters, protein absorption and retention than the ones fed cowpea diets (*p* < 0.05; Table 3). No difference in apparent or true protein R/A was found between rats fed casein control and raw cowpea diets, whereas lower values were found in both fermented cowpea diets (*p* < 0.05).

#### 3.2.2. Protein and Amino Acids Digestibility

The apparent and true fecal digestibility (AFD and TFD) of crude protein and amino acids is presented in Table 4 and Table 5. AFD and TFD of protein was lower in cowpea diets compared to the control diet (*p* < 0.05). Except for Cys (*p* > 0.05), control diet showed greater AFD for all amino acids (*p* < 0.05) compared to raw cowpea diet. Similarly, fermented and fermented plus autoclaved cowpea diets had lower AFD for all amino acids except for His (*p* > 0.05) compared to the control diet. Not considering methionine, supplemented to all diets, Arg and His had the greatest digestibility amongst indispensable amino acids of cowpea diets. The amino acid with the lowest AFD was Lys for RV and Ile for control, FV and FAV diets. Fermentation increased AFD of Leu, Lys, Met, Phe, Tyr, and Val compared to raw cowpea diets (*p* < 0.05), whereas autoclaving of fermented cowpea decreased AFD of sulfur amino acids and increased AFD of Ala and Pro compared to FV (*p* < 0.05).

True fecal digestibility (TFD) of amino acids was greater in the casein control compared to cowpea diets (*p* < 0.05), except for Arg, Gly, and Cys, where no differences were found (*p* > 0.05). Arg had the greatest TFD among the indispensable amino acids of cowpea. The lowest TFD was found for Ile in control, and FV, FAV and Lys for RV diets, respectively. Fermentation increased TFD of His, Leu, Lys, Met, Phe, Tyr, and Val compared to raw cowpea diets (*p* < 0.05). Autoclaving fermented cowpea decreased Met and Cys and increased Pro TFD compared to fermented cowpea diet (*p* < 0.05) with no further changes in other amino acids.

#### 3.2.3. Protein Quality Indices

All the experimental diets provided a sufficient amount of essential amino acids to meet the requirements of preschool children according to FAO/WHO/UNU recommendations (CS > 100%). However, when true protein digestibility values were used to correct the CS, the protein index value was markedly reduced in cowpea diets. Since Met was supplemented to all diets, Leu was the first limiting amino acid and so it was used to calculate PDCAAS (115 vs. 96.7 in casein control and RV, respectively). Fermentation or fermentation plus autoclaving did not significantly alter protein quality indices compared to the RV diet (data not shown).

#### 3.2.4. Mineral Bioavailability

##### Phosphorus and Calcium

The effects of natural fermentation combined or not with autoclaving on the digestive and metabolic utilization of P and Ca are presented in Table 6. Daily P intake and P fecal excretion was significantly higher in rats fed the different cowpea diets compared to the casein control. The highest P fecal excretion value was found for the RV group, whereas no differences were observed between the fermented Vigna diets, giving rise to a significantly lower digestive utilization of P in RV vs. control group. A significant improvement in P digestibility was achieved by fermentation, although with lower values than the control diet. No significant differences were found in urinary P excretion among the control or experimental groups. Retained P in rats fed RV diet was the lowest observed (*p* < 0.05), and no differences were found in the rest of experimental groups. However, metabolic utilization of P assessed as percentage of retained relative to absorbed mineral did not differ significantly among the dietary groups.

Although no significant differences in Ca intake were observed among the experimental groups, fecal excretion of this mineral was higher (*p* < 0.05) in animals fed Vigna vs. casein control diet, thus resulting in lower net absorption and digestive utilization in the former experimental groups, especially for FV and FAV. In contrast, fermentation and fermentation combined with autoclaving of Vigna resulted in lower urinary excretion of Ca compared to raw Vigna and control rats. In fact, the FAV and control groups showed the highest metabolic utilization of this mineral when expressed as ratio of retained relative to absorbed values. Nevertheless, net Ca retention was lower in animals fed cowpea vs. control diet.

##### Magnesium and Potassium

The effects of natural fermentation combined or not with autoclaving on the digestive and metabolic utilization of Mg and K are presented in Table 7. Digestive utilization of Mg and K was characterized by the greater Mg and K intake and Mg fecal excretion by the cowpea compared to the casein diet, with the highest Mg intake found for the RV group. Net absorption of Mg and K was also greater for cowpea groups compared to control group although no major differences in digestive utilization expressed as apparent fecal digestibility were found except for the lower Mg AFD of group FAV. Urinary excretion of Mg and K was significantly higher for the animals fed cowpea experimental diets giving rise to similar final net retention for all the experimental groups and considerably lower metabolic utilization of the two minerals among the cowpea groups, expressed as percentage of retained to absorbed mineral.

#### 3.2.5. Mineral Content in Tissues and Organs

Despite the above described differences in the digestive and metabolic utilization of minerals; no apparent differences in mineral content of blood, femur, and *longissimus dorsi* muscle were observed under our experimental conditions (Table 8), with the exception of P content in *longissimus dorsi* muscle and Ca content in femur that were slightly lower in the animals fed cowpea diets.

## 4. Discussion

Cowpea is one of the most important cultivated legumes, showing interesting agronomic and environmental benefits, as well as great potential as a nutritious and healthy food. Although it has been reported that cowpea has a promising nutrient composition, especially regarding protein and mineral content, few studies have focused on its in vivo digestibility, in relation to the legume potential as functional food. The aim of this study was to assess the effects of the biotechnological process of fermentation combined or not with autoclaving of *Vigna unguiculata* flour on the protein quality, amino acid digestibility, and bioavailability of four essential minerals using the rat as experimental model. Our results showed that cowpea provides a significant amount of digestible essential amino acids and fermentation improved their digestibility, making this legume a good source of highly available protein, although still inferior than casein. In addition, *Vigna unguiculata* was a good source of available P, Mg, and K and fermentation significantly enhanced the availability of P. The combination of fermentation with autoclaving did not enhance mineral availability of the fermented *V unguiculata* with the exception of the metabolic utilization of Ca.

The fermentation process selected for this experiment has been carried out based on the study of Doblado et al. [12] who assayed different bean flour concentrations and fermentation times with the aim of achieving optimal fermentation conditions to improve the nutritional quality of *Vigna unguiculata*. The authors reported a significant decrease in the content of antinutritional factors (TIA, inositol phosphates, and α-galactosides) and higher riboflavin content caused by fermentation, among other changes. When fermentation was combined with autoclave treatment, a further reduction in TIA was attained. Such reduction in antinutritional factor content more likely played a significant role in the higher digestibility of indispensable amino acids and P observed under the experimental conditions of the present study. Furthermore, the benefits of this same fermentation process on the functional value of *Vigna unguiculata* were studied by Kapravelou et al. [4], who reported a significant improvement in antioxidant capacity and different parameters of lipid metabolism induced by fermented beans in an in vivo rat experimental model.

The crude protein and mineral content of cowpea flours was within the range of values reported in the literature [6,27] for numerous cowpea cultivars. Patterns of amino acid composition were similar to those reported by previous studies in cowpea [27,28]. Although Granito et al. [29] found lower protein content of the cowpea cultivar Orituco after fermentation, other authors reported no alteration of protein or mineral content in cowpea seeds [2,30], as in the present study. Fermentation or the combination of fermentation and autoclaving did not substantially alter the amino acid profile of cowpea, being similar to what has been reported for germinated cowpea flour [31]. The increase of the time of thermal treatment has been reported to reduce protein content [32] although this was not confirmed by our study.

Regarding the mineral content of the experimental *V. unguiculata* diets, P and Mg were mainly provided by the cowpea flours, while only 43% of K was supplied by the flour. Nevertheless, due to the high solubility of K from legumes under similar conditions to those present in the gastrointestinal tract of monogastrics, K from cowpea was easily exchangeable with that from the mineral premix in the intestinal lumen. In contrast, close to 95% of Ca came from external sources such as CaHPO_4_ or the mineral premix. Although fermentation does not cause major changes in total mineral content of legume flours, it may affect the form in which they are present, therefore affecting their availability [33]. Specifically, it can increase the acidity of flours through the release of organic acids that may form mineral complexes and affect their solubility. In addition, fermentation process improves mineral availability by minimizing the action of non-nutritional components with known inhibitory effect on mineral digestibility including phytic acid or polyphenols [2]. Mineral content and availability are also decreased by thermal treatment [32], although in the present study fermentation combined with thermal treatment did not cause any further reduction. Under our experimental conditions, protein intake in growing rats fed cowpea diets was somewhat lower than in rats fed a casein diet of similar crude protein concentration. Conversely, a deep depression of protein intake in rats fed non supplemented raw cowpea for four weeks compared to a casein diet has been reported [34]. Low dietary intake of legume-based diets may be related to the presence of antipalatable compounds (α-galactosides or tannins) and deficiencies in certain indispensable amino acids (mainly Met), minerals, and vitamins, leading to nutrient imbalance. The effect of autoclave treatment on non-palatable factors is variable [35] although it did not seem to play a major role on dietary intake in the present study. In our experiment, cowpea fermentation and autoclaving did not alter dietary or protein intake, a finding that may indicate that cowpea cultivar used had low levels of antipalatable factors or that feeding duration was not long enough to detect subtle changes on dietary intake.

Growth of rats fed cowpea diets was adequate although lower than that of rats fed the casein diet. The satisfactory growth of the rats fed raw cowpea diet under our experimental conditions might be explained by a low amount of non-nutritional factors and the balancing of the amino acid profile due supplementation with the first limiting amino acid. In contrast, rats fed raw cowpea diets for 28 days [34] lost weight and exhibited negative protein efficiency ratio (PER).

We studied the metabolic utilization of protein as nitrogen retention and R/A after ad libitum consumption of the experimental diets. Nitrogen retention should be considered in conjunction with other indices such as PER and FTI. Under our experimental conditions, higher N intake and digestibility of control diet might explain the differences in growth observed compared to cowpea diets. True nitrogen R/A was similar in control compared to raw cowpea diet in spite of higher N absorption and similar endogenous urinary N. However, the metabolic utilization of protein from cowpea diets was much greater than in rats fed other legumes [36,37], a finding that is reflected in our experiment as a greater nutritive utilization of protein and its direct use mainly for plastic growth functions as opposed to other secondary uses that finally result in a lower rate of metabolic use. 

Legumes show protein digestibility ranging from 70% to 80% [38] in accordance with the results presented herein. Nevertheless, true protein digestibility of diets formulated with cowpea [29] and cowpea protein isolate [39] was greater than the ones reported in the present experiment, a finding probably related to methodological differences in endogenous protein estimation, since we used a low protein diet, while the former authors used protein-free diets. High non-starch polysaccharide content of the cowpea diets used in our study (86.6 mg g^−1^) may be related to their low protein digestibility, as high N excretion values are typically linked to high dietary non starch polysaccharide content in single-stomached animals [40,41]. Additionally, contribution of intestinal microbiota to fecal N excretion may be an additional factor to consider. It has been reported that bacterial N accounts for 50–80% of the total fecal N in fava bean or chickpea-fed rats [42]. Indeed, total bacterial counts greatly increased in feces of rats fed cowpea diets compared to a casein diet [43].

Amino acid digestibility of cowpea protein is usually determined by in vitro methods [44]. However, information about in vivo amino acid digestibility of cowpea protein is scarce. In this regard, cockerels fed unsupplemented cowpea flour had similar apparent fecal digestibility of indispensable amino acids compared to the present study [45]. Under our experimental conditions, natural fermentation improved apparent and true fecal digestibility of numerous indispensable amino acids which may be explained by diminished concentration of protease inhibitors, as it has been reported that fermentation almost eliminated trypsin inhibitor activity and lowered phytic acid and polyphenol content, main non-nutritional factors in cowpeas [29,46]. Heat treatment procedures have proved to be adequate methods for reducing contents or activity of several secondary plant metabolites in legumes [47], especially those of the heat labile group (protease inhibitors and lectins). Furthermore, as shown in peas, heat treatment technologies may induce conformational changes in storage proteins, which may render them more accessible to digestive enzymes, and thus may increase amino acid digestibility. In our experiment, autoclaving of fermented cowpea flour did not further increase the digestibility of amino acid, but decreased the apparent and true digestibility of sulfur amino acids. This fact could be due to the degradation of methionine by the Maillard reactions, during food processing [48] decreasing its availability [49].

The improved digestibility of essential amino acids achieved by fermentation was not reflected, under the experimental conditions of the present study, in higher growth parameters, such as the weight gain, or protein nutritional indices, such as PER or FTI, compared to the raw cowpea protein. This lack of correlation can be attributed to the experimental design in which supplementation of methionine to legume diets partially hindered the benefits of fermentation in amino acid digestibility, or else to the fact that differences in amino acid digestibility among raw and fermented cowpea were not sufficiently high to result in higher growth indices, a potential limitation of our study. In addition, the specific structure of legume proteins and the presence in legume diets of non-nutritional components that may affect protein digestion and absorption may also be responsible for this different behavior, compared to the animal-derived casein control protein, in which a higher protein and amino acid digestibility led to significantly higher growth and nutritive utilization indices. Such different behavior can also be attributed to the higher content of highly available wheat starch incorporated to the casein control, compared to the cowpea diets, in which a considerable proportion of the starch was legume-derived and has been described to be less available. Finally, although the fermentation protocol was optimized to reduce the content of non-nutritional components that interfere with protein digestibility, it did not cause major improvements in the amino acid profile compared to raw cowpea protein, thus minimizing the positive effects of a greater amino acid digestibility.

Nutritive utilization of the minerals studied appeared to be affected by their concentration in the experimental diets, possible interactions with the food matrix or distinctive bioavailability regulation at the digestive and urinary level. Digestive and metabolic utilization of minerals from plant-based foods is usually affected by protein quality, the presence of dietary fiber and non-nutritional components, such as phytic acid or polyphenols that may interfere with absorption [2]. The former inhibitory effects can be improved by biotechnological treatments like germination or fermentation that are able to generate new dietary components, such as organic acids capable of solubilizing and improving mineral absorption [33]. In the present experiment, we tried to equilibrate the potential effect of legume dietary fiber, formulating a casein control with similar amounts of the fiber components found in *V. unguiculata*. Such modifications in the casein diet resulted in a lower ratio of dietary intake/fecal excretion compared to *V. unguiculata* diet, thus indicating a higher proportion of fiber in feces of casein control and lower degree of gut fermentation. Nevertheless, no apparent relationship was found between fecal weight, which is a measure of the mineral dragging action of dietary fiber, and mineral excretion. On the other hand, the amount of ingested mineral can affect its digestibility with lower digestive utilization in response to increasing intake. In addition, P and Ca bioavailability appeared to be affected by other dietary factors. In the case of Ca, despite a similar mineral source in the diet and daily intake, digestive utilization from *V. unguiculata* diets was inferior to that from the casein control and did not improve as a result of fermentation process in a similar way to what has been detected in total protein. Although fermentation can reduce the amount of non-nutritional components present in legumes, such as phytic acid and polyphenols, to modify the structure of dietary fiber, and to release factors than enhance mineral bioaccessibility, such as organic acids, such changes were not sufficient to improve Ca bioavailability under our experimental conditions. An improvement regarding P availability likely related to fermentation was the reduction in phytic acid content and the release of potentially available P [2,50]. With regard to Mg and K, *V. unguiculata* was a source of highly available minerals. In the case of Mg, digestibility was mainly affected by its high dietary intake, whereas the effect of other legume flour components, such as phytic acid or polyphenols, appeared to be minor as seen by the lack of differences between raw or fermented *V. unguiculata*. The digestibility of K was extremely high under the experimental conditions of the present study, thus confirming the extraordinary potential of legumes as excellent dietary sources of this mineral with comparable availability to that of currently used dietary or pharmacological supplements. The nutritional importance of K is of outmost importance due to its participation in numerous cell functions, its protective action against kidney stone formation, and its essential role in bone health and in the regulation of blood pressure.

An interesting finding of this research is the different metabolic regulation of the minerals studied. In this regard, P metabolism was mainly regulated at the digestive level. Since Ca levels in the diet were adequate, most of absorbed P was retained in the body and the urinary reabsorption mechanisms worked very efficiently to achieve a mineral retention similar to that of the casein control diet. This finding was particularly evident in the groups fed fermented cowpea, in which the amount of mineral absorbed was enhanced by the technological treatment. On the other hand, K appeared to be regulated mainly at the urinary excretion level since nearly all the dietary ingested K was absorbed. Such urinary regulation bears two important facts: first, net retention of the mineral was similar for all treatments. Second, the higher urinary excretion of this mineral is reflected in changes in pH and urinary composition with well-known beneficial action to prevent kidney stone formation. Finally, Mg regulation appeared to take place at the digestive and urinary level to achieve similar net retention of the mineral compared to the casein control. With regard to Ca, and due to the adequate bioavailability of P from the diets, metabolic utilization of Ca was high, although not sufficient to reach the net retention values of the casein control. Such inferior retention values paralleled those of total N retention and body weight gain, and resulted in slightly lower levels of the mineral in femur bone and *longissimus dorsi* muscle.

## 5. Conclusions

Cowpea is a valuable foodstuff that can be used as nutritious functional ingredient. In addition to its health-related properties, the nutrient availability from this legume showed great potential, as seen by the high protein quality of raw and fermented cowpea after supplementation with methionine, reaching levels close to those of a high-quality reference protein. Such promising nutritive utilization of protein was matched by a high availability of essential minerals like P, Mg and K. Fermentation was efficient at improving the digestibility of most indispensable amino acid and P, although growth indexes were not affected. Autoclaving did not further increase the digestibility and growth parameters of fermented cowpea flour.

## Figures and Tables

**Table 1:** Composition of the experimental diets.

Diet ^a^	LPD4%	Control	RV	FV	FAV
**Ingredients (g·kg^−1^)**					
Casein	45.5	136.0	-	-	-
Cowpea flour	-	-	500.0	500.0	500.0
Methionine	5.0	5.0	5.0	5.0	5.0
Olive oil	70.0	70.0	70.0	70.0	70.0
Cellulose	50.0	26.0	-	-	-
Agar	-	27.0	-	-	-
Oat xylan	-	4.4	-	-	-
Potato starch	-	16.0	-	-	-
Lignin	-	13.0	-	-	-
Citrus pectin	-	38.5	-	-	-
Sucrose	100.0	100.0	100.0	100.0	100.0
Mineral mix	35.0	35.0	35.0	35.0	35.0
Vitamin mix	10.0	10.0	10.0	10.0	10.0
Choline bitartrate	2.5	2.5	2.5	2.5	2.5
Calcium Citrate	24.0	24.0	19.2	19.2	19.2
CaHPO_4_	-	-	7.5	7.5	7.5
Wheat starch	658.0	495.0	250.0	250.0	250.0
**Chemical Composition (In Dry Matter)**					
Total N (g·kg^−1^)		20.8	19.0	19.6	19.0
Total amino acids (g·kg^−1^)		132	107	104	101
Ash (g kg^−1^)		35.7	41.0	42.0	42.5
Ca (mg kg^−1^)		4806	5126	5283	5597
P (mg·kg^−1^)		3025	4006	4302	4317
Mg (mg·kg^−1^)		711	1165	1027	1014
K (mg·kg^−1^)		4274	7345	7796	7322

^a^ LPD4%, low protein diet; control, casein diet; RV, raw cowpea; FV, fermented cowpea; FAV, fermented and autoclaved cowpea. Mineral mix added to *Vigna unguiculata* diets lacked sources of P, Ca, and Mg, and had a sufficient amount of K to meet the nutrient requirements of a growing rat taking in consideration the amount of this mineral provided by raw or fermented *Vigna unguiculata* flours.

**Table 2:** Amino acid profile of egg protein and experimental diets expressed in g/16 g N and amino acid content (g·kg^−1^) of the experimental diets.

Diet ^a^	Egg	Control	RV	FV	FAV
**Indispensable**					
Arginine	6.0	4.75 (6.26)	9.09 (9.76)	9.35 (9.75)	8.59 (8.68)
Histidine	2.2	2.96 (3.90)	4.21 (4.53)	4.17 (4.35)	4.58 (4.62)
Isoleucine	5.4	3.89 (5.12)	4.80 (5.15)	4.47 (4.66)	4.33 (4.37)
Leucine	8.6	10.60 (14.0)	7.68 (8.24)	7.42 (7.74)	7.42 (7.50)
Lysine	7.2	11.47 (15.1)	7.08 (7.61)	7.74 (8.07)	7.90 (7.98)
Methionine	5.7 (Met + Cys)	3.23 (4.25)	3.68 (3.95)	3.67 (3.83)	3.63 (3.67)
Phenylalanine	9.3 (Phe + Tyr)	6.26 (8.24)	6.76 (7.26)	5.85 (6.10)	5.76 (5.82)
Tyrosine	-	4.59 (6.04)	4.21 (4.52)	4.85 (5.06)	4.39 (4.44)
Threonine	4.7	4.38 (5.77)	4.80 (5.15)	4.05 (4.23)	4.39 (4.44)
Valine	6.6	5.62 (7.40)	6.25 (6.71)	5.58 (5.82)	5.31 (5.37)
BCAA	20.6	20.1 (26.5)	18.7 (20.1)	17.5 (18.2)	17.1 (17.2)
Total indispensable	55.7	57.4 (76.1)	58.6 (62.9)	57.2 (59.6)	56.3 (56.9)
**Dispensable**					
Alanine	-	4.45 (5.86)	4.95 (5.32)	4.77 (4.98)	4.02 (4.06)
Aspartate	-	6.40 (8.43)	7.3 (7.87)	8.99 (9.38)	9.71 (9.82)
Cysteine	-	0.61 (0.80)	0.91 (0.98)	1.00 (1.04)	0.92 (0.93)
Glutamate	-	16.73 (22.02)	14.91 (16.01)	13.44 (14.01)	14.23 (14.39)
Glycine	-	2.15 (2.83)	4.35 (4.68)	3.83 (4.00)	4.06 (4.10)
Proline	-	8.13 (10.70)	5.15 (5.53)	6.26 (6.53)	6.20 (6.26)
Serine	-	3.78 (4.98)	3.85 (4.14)	4.54 (4.73)	4.56 (4.61)

^a^ Control: casein diet; RV: raw cowpea; FV: fermented cowpea; FAV: fermented and autoclaved cowpea; BCAA: branched chain amino acids.

**Table 3:** Nutritive utilization of protein in rats (*n* = 10) fed diets based on casein or cowpea as the only protein sources.

Diets ^a^	Control	RV	FV	FAV
N Intake (mg/d)	276 (5.6) ^B^	239 (5.82) ^A^	244 (5.7) ^A^	248 (7.5) ^A^
Total fecal N (mg/d)	30 (0.9) ^A^	57 (1.6) ^B^	54 (2.2) ^B^	57 (2.4) ^B^
Endogenous fecal N (mg/d)	6.7 (0.4) ^AB^	8.0 (0.4) ^B^	5.8 (0.3) ^A^	7.8 (0.9) ^AB^
Apparent absorbed N (mg/d)	246 (5.0) ^B^	182 (4.9) ^A^	189 (4.4) ^A^	191 (5.9) ^A^
True absorbed N (mg/d)	252 (5.1) ^B^	190 (5.1) ^A^	195 (4.5) ^A^	198 (6.3) ^A^
Total urinary N (mg/d)	49 (2.5)	42 (2.0)	50 (2.7)	51 (3.8)
Endogenous urinary N (mg/d)	21 (1.8)	25 (1.7)	21 (1.3)	21 (1.2)
Apparent retained N (mg/d)	197 (3.8) ^A^	140 (4.7) ^B^	140 (3.7) ^B^	140 (6.0) ^B^
True retained N (mg/d)	225 (3.8) ^B^	173 (4.7) ^A^	164 (3.8) ^A^	168 (5.2) ^A^
Apparent R/A	0.80 (0.008) ^B^	0.77 (0.006) ^AB^	0.74 (0.012) ^A^	0.74 (0.014) ^A^
True R/A	0.89 (0.01) ^B^	0.91 (0.01) ^B^	0.85 (0.01) ^A^	0.85 (0.02) ^A^

^a^ Control: casein diet; RV: raw cowpea diet; FV: fermented cowpea diet; FAV: fermented and autoclaved cowpea diet; R/A: Retention to Absorption Index ({[*I* − (*F* + *U*)]/(*I* − *F*)}, where *I* = intake, *F* = fecal excretion, and *U* = urinary excretion). Data are expressed as means and standard error of the mean (SEM), (in parenthesis). Means within a row with different superscripts differ significantly (*p* < 0.05).

**Table 4:** Apparent fecal digestibility, g·kg^−1^ of amino acids in rats (*n* = 10) fed diets based on casein or cowpea as the only protein sources.

Diets ^a^	Control	RV	FV	FAV
**Indispensable**				
Arginine	885 (7.2) ^B^	834 (8.3) ^A^	860 (6.6) ^AB^	835 (9.6) ^A^
Histidine	892 (4.2) ^B^	837 (26.8) ^A^	847 (8.4) ^AB^	839 (9.2) ^AB^
Isoleucine	878 (3.9) ^B^	779 (10.0) ^A^	785 (9.8) ^A^	762 (12.6) ^A^
Leucine	939 (1.5) ^C^	756 (11.5) ^A^	810 (9.3) ^B^	791 (10.6) ^B^
Lysine	928 (1.8) ^C^	692 (14.6) ^A^	811 (10.6) ^B^	798 (12.2) ^B^
Methionine	936 (3.0) ^B^	892 (10.4) ^A^	965 (1.3) ^D^	948 (2.4) ^C^
Phenylalanine	938 (2.7) ^C^	724 (11.9) ^A^	808 (9.7) ^B^	790 (10.4) ^B^
Tyrosine	894 (3.8) ^C^	774 (10.9) ^A^	834 (8.4) ^B^	802 (10.8) ^AB^
Threonine	917 (3.8) ^B^	820 (8.5) ^A^	797 (12.1) ^A^	791 (12.0) ^A^
Valine	907 (2.5) ^C^	751 (10.6) ^A^	808 (8.8) ^B^	795 (10.9) ^B^
**Dispensable**				
Alanine	892 (2.7) ^C^	729 (14.1) ^A^	790 (10.2) ^B^	728 (18.8) ^A^
Aspartate	903 (4.3) ^C^	803 (10.5) ^A^	896 (5.2) ^B^	897 (8.3) ^B^
Cysteine	759 (12.3) ^AB^	727 (25.7) ^A^	792 (8.3) ^B^	747 (11.8) ^A^
Glutamate	939 (2.1) ^B^	835 (8.0) ^A^	845 (6.8) ^A^	843 (9.5) ^A^
Glycine	805 (8.2) ^B^	758 (10.8) ^A^	778 (11.1) ^AB^	765 (14.0) ^AB^
Proline	951 (2.3) ^D^	739 (12.9) ^A^	793 (11.6) ^B^	839 (6.8) ^C^
Serine	904 (3.4) ^B^	799 (9.3) ^A^	816 (9.9) ^A^	800 (11.4) ^A^
Sum of amino acids	920 (2.4) ^C^	804 (8.8) ^A^	843 (7.2) ^B^	836 (8.9) ^B^
Protein	891 (2.4) ^B^	761 (4.0) ^A^	771 (7.9) ^A^	787 (9.0) ^A^

^a^ Control: casein diet; RV: raw cowpea diet; FV: fermented cowpea diet; FAV: fermented and autoclaved cowpea diet. Data are expressed as means and standard error of the mean (SEM), (in parenthesis). Means within a row with different superscripts differ significantly (*p* < 0.05).

**Table 5:** True fecal digestibility, g·kg^−1^ of amino acids in rats fed diets based on casein or cowpea as the only protein sources.

Diets ^a^	Control	RV	FV	FAV
**Indispensable**				
Arginine	968 (6.9) ^B^	922 (8.5) ^A^	942 (6.8) ^AB^	922 (10.1) ^A^
Histidine	953 (3.9) ^C^	868 (26.2) ^A^	911 (8.4) ^B^	907 (9.6) ^B^
Isoleucine	921 (4.0) ^B^	830 (10.0) ^A^	829 (9.9) ^A^	808 (12.9) ^A^
Leucine	970 (1.6) ^C^	788 (11.5) ^A^	841 (9.4) ^B^	823 (10.8) ^B^
Lysine	962 (1.9) ^C^	728 (14.6) ^A^	845 (10.6) ^B^	834 (12.4) ^B^
Methionine	1004 (2.7) ^D^	961 (8.5) ^A^	990 (1.3) ^C^	974 (2.6) ^B^
Phenylalanine	973 (2.7) ^C^	761 (11.8) ^A^	841 (9.8) ^B^	826 (10.6) ^B^
Tyrosine	928 (4.1) ^C^	810 (11.0) ^A^	868 (8.5) ^B^	838 (11.0) ^AB^
Threonine	973 (3.7) ^B^	880 (8.5) ^A^	853 (12.1) ^A^	850 (12.4) ^A^
Valine	951 (2.6) ^C^	808 (10.7) ^A^	852 (9.0) ^B^	841 (11.1) ^AB^
**Dispensable**				
Alanine	956 (2.6) ^C^	856 (14.4) ^B^	855 (10.2) ^B^	796 (19.2) ^A^
Aspartate	936 (4.3) ^B^	839 (10.5) ^A^	931 (5.4) ^B^	935 (8.7) ^B^
Cysteine	940 (9.5) ^C^	929 (25.4) ^C^	844 (8.4) ^B^	801 (12.0) ^A^
Glutamate	986 (2.0) ^B^	884 (8.0) ^A^	891 (7.0) ^A^	893 (9.9) ^A^
Glycine	873 (8.0)	829 (10.8)	845 (11.1)	835 (14.4)
Proline	958 (2.2) ^D^	747 (12.9) ^A^	801 (11.6) ^B^	847 (6.8) ^C^
Serine	983 (3.3) ^B^	882 (9.4) ^A^	895 (10.0) ^A^	884 (11.9) ^A^
Sum of amino acids	966 (2.4) ^C^	852 (8.7) ^A^	889 (7.3) ^B^	884 (9.2) ^B^
Protein	915 (2.3) ^B^	794 (5.8) ^A^	802 (6.9) ^A^	801 (5.8) ^A^

^a^ Control: casein diet; RV: raw cowpea diet; FV: fermented cowpea diet; FAV: fermented and autoclaved cowpea diet. Data are expressed as means and standard error of the mean (SEM), (in parenthesis). Means within a row with different superscripts differ significantly (*p* < 0.05).

**Table 6:** Effect of natural fermentation on the nutritive utilization of P and Ca from *Vigna unguiculata* diets.

Diets ^a^	Control	RV	FV	FAV
**Phosphorus**				
P Intake (mg/d)	40.1 (0.8) ^A^	50.6 (1.2) ^B^	52.9 (1.2) ^B^	54.7 (1.7) ^B^
Fecal Excretion (g/d)	1.30 (0.04) ^B^	1.01 (0.04) ^A^	0.98 (0.04) ^A^	1.09 (0.04) ^A^
Fecal P (mg/d)	5.99 (0.30) ^A^	23.8 (0.95) ^C^	19.8 (0.9) ^B^	20.6 (1.1) ^B^
Urinary P (mg/d)	0.47 (0.05)	0.94 (0.14)	0.31 (0.03)	0.28 (0.04)
Absorbed P (mg/d)	34.1 (0.6) ^B^	26.7 (0.5) ^A^	33.1 (0.97) ^B^	34.2 (0.90) ^B^
P AFD	0.85 (0.01) ^C^	0.53 (0.01) ^A^	0.63 (0.01) ^B^	0.63 (0.01) ^B^
Retained P (mg/d)	33.7 (0.6) ^B^	25.8 (0.5) ^A^	32.8 (1.0) ^B^	33.9 (0.9) ^B^
P R/A	0.99 (0.001)	0.97 (0.005)	0.99 (0.001)	0.99 (0.001)
**Calcium**				
Ca Intake (mg/d)	63.8 (1.3)	64.7 (1.6)	65.7 (1.5)	71.0 (2.1)
Fecal Ca (mg/d)	14.4 (0.6) ^A^	37.7 (1.4) ^B^	40.3 (2.5) ^BC^	45.6 (2.3) ^C^
Urinary Ca (mg/d)	9.14 (0.41) ^B^	8.56 (0.35) ^B^	5.81 (0.25) ^A^	4.92 (0.33) ^A^
Absorbed Ca (mg/d)	48.7 (1.0) ^B^	27.0 (1.23) ^A^	23.6 (1.8) ^A^	25.4 (1.3) ^A^
Ca AFD	0.76 (0.01) ^B^	0.42 (0.02) ^A^	0.37 (0.03) ^A^	0.36 (0.02) ^A^
Retained Ca (mg/d)	39.5 (1.0) ^B^	18.7 (1.0) ^A^	17.8 (1.7) ^A^	20.5 (1.2) ^A^
Ca R/A	0.81 (0.01) ^B^	0.69 (0.01) ^A^	0.74 (0.02) ^AB^	0.80 (0.01) ^B^

^a^ Control: casein diet; RV: raw cowpea diet; FV: fermented cowpea diet; FAV: fermented and autoclaved cowpea diet. AFD, Apparent Fecal Digestibility, R/A, Retained to absorbed ratio. Data are expressed as means and standard error of the mean (SEM), (in parenthesis). Means within a row with different superscripts differ significantly (*p* < 0.05).

**Table 7:** Effects of natural fermentation on the nutritive utilization of Mg and K from *Vigna unguiculata* diets.

Diets ^a^	Control	RV	FV	FAV
**Magnesium**				
Mg Intake (mg/d)	9.4 (0.2) ^A^	14.7 (0.4) ^C^	12.8 (0.3) ^B^	12.9 (0.4) ^B^
Fecal Mg (mg/d)	3.53 (0.13) ^A^	5.55 (0.19) ^B^	4.87 (0.21) ^B^	5.62 (0.23) ^B^
Urinary Mg (mg/d)	2.77 (0.16) ^A^	5.60 (0.25) ^C^	4.76 (0.30) ^BC^	4.33 (0.27) ^B^
Absorbed Mg (mg/d)	5.90 (0.17) ^A^	9.16 (0.27) ^C^	7.90 (0.21) ^B^	7.24 (0.20) ^B^
Mg AFD	0.63 (0.01) ^B^	0.62 (0.01) ^B^	0.61 (0.01) ^B^	0.56 (0.01) ^A^
Retained Mg (mg/d)	3.14 (0.15)	3.56 (0.08)	3.14 (0.31)	2.91 (0.22)
Mg R/A	0.53 (0.02) ^B^	0.39 (0.01) ^A^	0.40 (0.03) ^A^	0.40 (0.03) ^A^
**Potassium**				
K Intake (mg/d)	56.7 (1.2) ^A^	92.8 (2.3) ^B^	96.9 (2.3) ^B^	92.9 (2.8) ^B^
Fecal K (mg/d)	5.23 (0.67)	8.40 (1.05)	5.64 (0.81)	6.20 (0.70)
Urinary K (mg/d)	23.6 (0.90) ^A^	52.5 (3.1) ^B^	56.4 (4.0) ^B^	51.9 (3.4) ^B^
Absorbed K (mg/d)	51.5 (1.0) ^A^	84.4 (3.0) ^B^	91.3 (2.2) ^B^	85.7 (2.3) ^B^
K AFD	0.91 (0.01) ^A^	0.91 (0.01) ^A^	0.94 (0.08) ^A^	0.92 (0.01) ^A^
Retained K (mg/d)	27.9 (0.9)	31.9 (1.1)	32.9 (2.0)	33.7 (1.9)
K R/A	0.54 (0.01) ^B^	0.38 (0.02) ^A^	0.36 (0.03) ^A^	0.40 (0.03) ^A^

^a^ Control: casein diet; RV: raw cowpea diet; FV: fermented cowpea diet; FAV: fermented and autoclaved cowpea diet. AFD, Apparent Fecal Digestibility, R/A, Retained to absorbed ratio. Data are expressed as means and standard error of the mean (SEM), (in parenthesis). Means within a row with different letters differ significantly (*p* < 0.05).

**Table 8:** Effect of natural fermentation on the mineral content of different tissues.

Diets ^a^	Control	RV	FV	FAV
Femur Ash (%)	55.7 (0.7)	55.5 (0.5)	55.8 (0.4)	55.1 (0.4)
LD muscle Ash (%)	4.58 (0.18) ^AB^	4.29 (0.27) ^A^	5.33 (0.22) ^B^	4.52 (0.23) ^AB^
**P**				
Blood (mg·100 mL^−1^)	48.6 (1.4)	49.0 (0.6)	46.5 (2.6)	44.8 (1.5)
Femur (mg·g^−1^ Ash)	174.8 (1.0)	174.8 (1.6)	173.2 (0.7)	173.0 (0.7)
LD muscle (mg·g^−1^ Ash)	199.7 (1.5) ^B^	193.0 (1.3) ^A^	190.8 (1.3) ^A^	189.2 (2.2) ^A^
**Ca**				
Blood (mg·100 mL^−1^)	6.56 (0.70) ^B^	5.00 (0.18) ^A^	5.45 (0.44) ^AB^	5.66 (0.36) ^AB^
Femur (mg·g^−1^ Ash)	310.1 (2.7) ^B^	320.1 (4.5) ^B^	291.6 (7.9) ^A^	271.1 (5.4) ^A^
LD muscle (mg·g^−1^ Ash)	27.5 (7.8)	33.2 (7.5)	26.5 (5.9)	25.5 (6.3)
**Mg**				
Blood (mg·100 mL^−1^)	4.34 (0.14)	4.37 (0.07)	4.27 (0.22)	4.24 (0.13)
Femur (mg·g^−1^ Ash)	7.27 (0.11) ^A^	7.93 (0.10) ^B^	7.61 (0.04) ^AB^	7.52 (0.03) ^A^
LD muscle (mg·g^−1^ Ash)	22.5 (0.5) ^B^	21.6 (0.4) ^AB^	20.9 (0.5) ^AB^	20.0 (0.8) ^A^
**K**				
Blood (mg·100 mL^−1^)	207.8 (12.6)	197.6 (4.5)	197.9 (15.9)	224.2 (10.5)
Femur (mg·g^−1^ Ash)	9.94 (0.39)	11.08 (0.58)	9.66 (0.27)	11.24 (0.31)
LD muscle (mg·g^−1^ Ash)	290.7 (9.1)	287.2 (8.4)	286.2 (9.9)	277.0 (7.9)

^a^ Control: casein diet; RV: raw cowpea diet; FV: fermented cowpea diet; FAV: fermented and autoclaved cowpea diet. LD: longissimus dorsi. Data are expressed as means and standard error of the mean (SEM), (in parenthesis). Means within a row with different superscripts differ significantly (*p* < 0.05).

**Figure A1:**
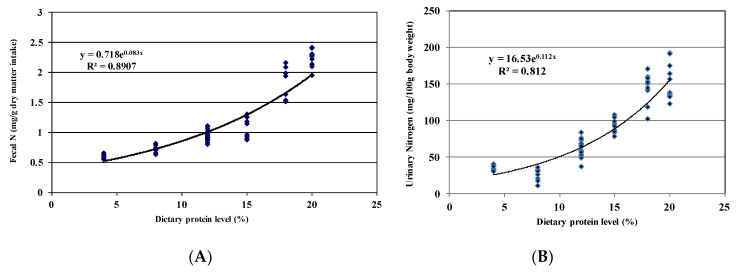
Fecal and urinary endogenous Nitrogen excretion calculated by a regression methodology using different dietary protein levels. (**A**) Endogenous fecal N excretion expressed as mg N per gram of dry matter intake. (**B**) Endogenous urinary excretion expressed as mg N per 100 g of body weight.

## Limitations of the Study

This in vivo study was conducted in rats. It should be validated in humans to fully explore the nutritional potential of the biotechnological treatment implemented. In addition, a control group with autoclaved non-fermented *Vigna unguiculata* was not included since the principal focus of the experiment was given to natural fermentation.
